# Structure–Function Analysis Reveals Amino Acid Residues of *Arabidopsis* Phosphate Transporter *At*PHT1;1 Crucial for Its Activity

**DOI:** 10.3389/fpls.2019.01158

**Published:** 2019-09-19

**Authors:** Ya-Yun Liao, Jia-Ling Li, Rong-Long Pan, Tzyy-Jen Chiou

**Affiliations:** ^1^Department of Life Science, Institute of Bioinformatics and Structural Biology, College of Life Science, National Tsing Hua University, Hsin Chu, Taiwan; ^2^Agricultural Biotechnology Research Center, Academia Sinica, Taipei, Taiwan

**Keywords:** phosphate transporter, AtPHT1;1, *Arabidopsis thaliana*, *Saccharomyces cerevisiae*, topology, site-directed mutagenesis, major facilitator superfamily

## Abstract

Phosphorus (P), an essential plant macronutrient, is acquired in the form of inorganic phosphate (Pi) by transporters located at the plasma membrane of root cells. To decipher the Pi transport mechanism, *Arabidopsis thaliana* Pi transporter 1;1 (*At*PHT1;1), the most predominantly H^+^-coupled Pi co-transporter in the root, was selected for structure–function analysis. We first predicted its secondary and tertiary structures on the basis of the *Piriformospora indica* Pi transporter (*Pi*PT) and identified 28 amino acid residues potentially engaged in the activity of *At*PHT1;1. We then mutagenized these residues into alanine and expressed them in the yeast *pam2* mutant defective in high-affinity Pi transporters and *Arabidopsis pht1;1* mutant, respectively, for functional complementation validation. We further incorporated the functional characterization and structure analyses to propose a mechanistic model for the function of *At*PHT1;1. We showed that D35, D38, R134, and D144, implicated in H^+^ transfer across the membrane, and Y312 and N421, involved in initial interaction and translocation of Pi, are all essential for its transport activity. When Pi enters the binding pocket, the two aromatic moieties of Y145 and F169 and the hydrogen bonds generated from Q172, W304, Y312, D308, and K449 can build a scaffold to stabilize the structure. Subsequent interaction between Pi and the positive residue of K449 facilitates its release. Furthermore, D38, D93, R134, D144, D212, R216, R233, D367, K373, and E504 may form internal electrostatic interactions for structure ensemble and adaptability. This study offers a comprehensive model for elucidating the transport mechanism of a plant Pi transporter.

## Introduction

Phosphorus (P) is one of the nutrients that are essential for all organisms. It is required for cellular constituents, including nucleic acids, membranes, and ATP, and is a key component in regulating enzyme reactions, metabolic pathways, and signal transduction processes (Chiou and Lin, 2011; Nussaume et al., 2011; Baek et al., 2017). Cells acquire P mainly in the form of inorganic phosphate (Pi, orthophosphate) by Pi transporters at plasma membranes. In plants, Pi is initially acquired by roots, moved toward the stele, transported up to the above-ground tissues, and subsequently distributed among different tissues. The acquisition and allocation of Pi mainly relies on the activities of plasma membrane-localized Pi transporters. Regulation of Pi transport activities by coordinating the environmental Pi supply with the demand of plants is a prerequisite to sustain plant growth and development and to ensure reproductive success.

The members of the phosphate transporter 1 (PHT1) family located at the plasma membranes are conserved in plant species and are responsible for Pi acquisition from the rhizosphere, as well as for Pi remobilization among different tissues (Nussaume et al., 2011; Johri et al., 2015). In *Arabidopsis thaliana*, nine genes (*AtPHT1;1* - *AtPHT1;9*) in the *PHT1* family were identified. Several of them are preferentially expressed in the root epidermal or cortical cells, supporting their roles in Pi uptake (Mudge et al., 2002; Poirier and Bucher, 2002). In particular, *At*PHT1;1 plays a predominate role in Pi acquisition under Pi replete conditions because *pht1;1* mutants showed less Pi accumulation than wild-type (WT) plants as a result of reduced Pi uptake activity (Shin et al., 2004; Ayadi et al., 2015). The *PHT1* genes are transcriptionally upregulated by Pi starvation and PHT1 proteins are post-translationally regulated *via* intracellular trafficking from the endoplasmic reticulum (ER) to the plasma membrane and ubiquitin-mediated degradation (González et al., 2005; Bayle et al., 2011; Huang et al., 2013; Lin et al., 2013). The Pi transport activities of several PHT1 members have been detected in transformed oocytes, yeast mutants, or plant cells (Muchhal et al., 1996; Mitsukawa et al., 1997; Ai et al., 2009). The Pi transport activity of *At*PHT1 was enhanced under low pH but inhibited by protonophores, indicating the nature of H^+^-coupled Pi symporters (Mitsukawa et al., 1997). Expression of *At*PHT1;1 in the BY-2 tobacco cell line exhibited a high-affinity Pi transport property (*K*_m_ = 3.1 μM) (Mitsukawa et al., 1997). Furthermore, it has been recently suggested that *At*PHT1;1 and *At*PHT1;4 could form a homomeric or heteromeric complex, which modulates Pi transport activity (Fontenot et al., 2015). Site-directed mutagenesis at Y312D residue of *At*PHT1;1 enhanced Pi transport likely due to the disruption of homomeric interactions (Fontenot et al., 2015). Despite the abovementioned work, our knowledge on the structure–function relationship of PHT1 proteins is still limited.

PHT1 proteins belong to the major facilitator superfamily (MFS) whose members could transport many kinds of small solutes (Pao et al., 1998). MFS represents the largest secondary transporter group using the electrochemical potential gradient generated by ATPase across membranes. They are typically 400 to 600 amino acids long consisting of 12 transmembrane (TM) α-helices and share similarities in TM topology with both N- and C-termini facing cytosol (Pao et al., 1998; Yan, 2015; Quistgaard et al., 2016). All MFS transporters contain a characteristic core folding, by which 12 TMs are divided into three groups depending on spatial arrangement at the plasma membrane, including the core helices (TMs 1, 4, 7, and 10), the middle helices (TMs 2, 5, 8, and 11), and the outer helices groups (TMs 3, 6, 9, and 12) (Yan, 2015). A general transport mechanism of MFS has been suggested based on the structure analysis of several representative members whose distinct conformational states include initial ligand-free occluded, outward open, ligand-bound occluded, and final inward open states (Yan, 2015; Quistgaard et al., 2016). These conformational states could be modulated by substrate interactions *via* hydrogen bonds and by the gating residues of A-motifs, GX_3_-(D/E)-(R/K)-X-G-[X]-(R/K)-(R/K), *via* internal salt bridges (Jiang et al., 2013; Quistgaard et al., 2016). Moreover, the A-motif between TM2 and TM3 in the N-domain and/or between TM8 and TM9 in the C-domain are/is able to form internal salt bridges with residues on TM5 or TM10 (Jiang et al., 2013; Quistgaard et al., 2016).

The phosphate:H^+^ symporter (PHS) family, belonging to MFS Family 9, has been identified in yeasts, fungi, and plants, including PHO84 from *Saccharomyces cerevisiae*, *Gv*PT from *Glomus versiforme*, and PHT1 members from plants (Bun-Ya et al., 1991; Harrison and van Buuren, 1995; Muchhal et al., 1996; Pao et al., 1998). The first crystal structure of a fungal Pi transporter *Pi*PT (*Piriformospora indica* PT) with an inward-facing ligand-bound occluded state was resolved (Pedersen et al., 2013). In *Pi*PT, several residues such as Y150, F174, Q177, W320, D324, Y328, N431, and K459 residues were found in the Pi binding pocket and D45, D48, E108, R139, and D149 residues have been suggested to be involved in H^+^ transfer (Pedersen et al., 2013). The transport mechanism of *Pi*PT was proposed to be mediated by conformational changes through protonation and deprotonation of D324 residue, triggering off an outward open state to a ligand-bound occluded state (Pedersen et al., 2013). The functional analysis of *Sc*PHO84 suggested the involvement of R168 and D178 residues (corresponding to R139 and D149 residues in *Pi*PT) in H^+^ transfer and Y179, D358, and K492 residues (corresponding to Y150, D324, and K459 residues in *Pi*PT) in the Pi-binding pocket (Samyn et al., 2012; Samyn et al., 2016). Nevertheless, the importance of these amino acid residues is not validated experimentally *in vivo*.

To further elucidate the structure–function relationship of Pi transporters, in this study, we were thus prompted to identify the key amino acid residues of *At*PHT1;1 engaged in the course of Pi transport, which shows protein sequence homology (30% identity and 48% similarity) with *Pi*PT. The selected candidate residues were subjected to alanine scanning mutagenesis, followed by functional complementation analysis in the yeast *pam2* mutant defective in two high-affinity Pi transporters and in *Arabidopsis pht1;1* mutants, respectively. Together with the structure information, our study offers experimental evidence to validate the importance of potential residues that may participate in Pi/H^+^ transport, maintain structural stability, or facilitate conformational changes. A working model is accordingly proposed to illustrate in a detailed manner the mechanism through which Pi transporters act.

## Materials and Methods

### Plant Materials and Growth Conditions

The *Arabidopsis thaliana* ecotype Columbia (Col-0) and a *pht1;1* T-DNA insertion line (SALK 088586) (Shin et al., 2004) obtained from the Arabidopsis Stock Center were used in this study. Seeds were surface sterilized and germinated for 5 days on agar plates with half-strength modified Hoagland’s nutrient solution containing 250 μM KH_2_PO_4_, 1% (w/v) sucrose, and 0.8% (w/v) Bacto-agar, and then transferred to high-Pi medium containing 1 mM KH_2_PO_4_, 1% (w/v) sucrose, and 1.2% (w/v) Bacto-agar for another 7 days.

### Plasmid Construction, Site-Directed Mutagenesis and Yeast Transformation

The full-length open reading frame of *AtPHT1;1* gene containing C-terminal His_6_-tag was cloned and recombined into the expression vector pYES2 (Invitrogen) through the *Hind*III and *Xba*I sites. Site-directed mutagenesis of *AtPHT1;1* was conducted based on QuikChange PCR methods (Kirsch and Joly, 1998). Each mutation was confirmed by sequencing. Sequences of primers used for cloning are listed in **Table S1**. WT *AtPHT1;1* and its mutation variants were transformed into *Saccharomyces cerevisiae pam2* (MATa pho3-1 Δ*pho84*::HIS3, Δ*pho89*::TRP1 ade2 leu2-3, 112 his3-532 trp-289 ura3-1,2 can1) (Martinez and Persson, 1998) using the LiAc/polyethylene glycol method (Gietz et al., 1995). As controls, an empty vector was transformed into BY4741 (MATa his3Δ1 leu2Δ0 met15Δ0 ura3Δ0) (Jensen et al., 2003) and *pam2*, respectively.

### *Arabidopsis* Transformation

The full-length open reading frame of WT *AtPHT1;1* and its mutation variants were cloned into the pCR8/GW/TOPO entry vector (Invitrogen) and validated by sequencing. All variants were recombined into the destination vector of pMDC32 *via* LR Clonase enzyme mix (Invitrogen), in which the cauliflower mosaic virus (CaMV) *35S promoter* (p35S) was replaced with *AtPHT1;1* promoter (3319 bp containing 5′ UTR and the first intron). The WT and individual variant of *AtPHT1;1* driven by its native promoter were introduced into the *pht1;1* mutant by an *Agrobacterium tumefaciens* (strain GV3101) dipping method (Clough and Bent, 1998). Transgenic plants were selected by 20 μg/ml hygromycin. For each variant, 8-10 independent T2 lines were examined.

### Measurement of Phosphate Contents

The 12-day-old seedlings were harvested and used for Pi contents analysis. Tissues were homogenized with 1% (v/v) glacial acetic acid and incubated at 42°C for 30 min. After centrifugation, the supernatant was collected and mixed with assay solution containing 0.35% (w/v) NH_4_MoO_4_, 0.86 N H_2_SO_4_, and 1.4% (w/v) ascorbic acid at 42°C for 30 min. Pi contents were determined by colorimetric measurement at OD_820_ based on the formation of phosphomolybdate followed by its reduction with ascorbic acid (Chiou et al., 2006).

### *Agrobacterium*-Mediated Infiltration of Tobacco Leaves

To observe the subcellular localization of *At*PHT1;1 variants, their C termini were fused with YFP and expressed in tobacco (*Nicotiana benthamiana*) leaves *via Agrobacterium*-mediated infiltration method according to the previous report (Lin et al., 2013). The fluorescence of YFP-tagged *At*PHT1;1 variants was observed at 3 days after infiltration by confocal microscopy.

### Yeast Manipulation, Growth Complementation, and Pi Transport Assay

Synthetic complete (SC) medium is composed of 5.7 g/L YNB (pH 4; Qbiogene), 0.77 g/L CSM-Ura (Qbiogene), 0.04 g/L adenine, 20 g/L d-glucose (d-Glu, non-induced medium) or d-galactose (d-Gal, induced medium) as the carbon source. Yeast cells were first grown to the logarithmic phase in SC medium containing high Pi (10 mM KH_2_PO_4_) plus 2% (w/v) d-Glu. Cells were washed with sterile water and resuspended with SC medium without Pi, and the cell density was adjusted to OD_600_ = 1.0. For complementation analysis, cells were diluted serially (from 10^−1^ to 10^−4^ fold) and 5 μl of cells were spotted onto SC medium plates containing 2% (w/v) Bacto-agar and high (10 mM KH_2_PO_4_) or low Pi (25 μM KH_2_PO_4_) supplied with either 2% (w/v) d-Glu or d-Gal, respectively. Plates were then incubated at 30°C for 7 days. To prepare for Pi transport assay, yeast was cultured in SC medium containing high Pi (10 mM KH_2_PO_4_) plus 2% (w/v) d-Glu overnight. After washing with sterile water, cells were adjusted to a density of OD_600_ = 0.05 and then grew in SC medium with 2% (w/v) d-Gal but without Pi at 30°C for 24 h. The yeast cell density was measured and Pi transport activity determined at 20 min after addition of [^33^P]KH_2_PO_4_ (a final concentration of 100 μM). The cells were immediately filtered and washed with 5-ml SC without Pi. The radioactivity of cells was measured by a liquid scintillation counter. The transport activity was normalized with the expressed *At*PHT1;1 protein amount and calculated relative to the WT control.

### Isolation of Membrane Proteins From Yeast

Yeast cells were grown in SC induced medium without Pi and harvested after 24 h. The pellet was resuspended in lysis buffer containing 100 mM Tris-Mes (pH 8.0), 1% (w/v) yeast extract, 2% (w/v) peptone, 1% (w/v) D-Glu, 0.7 M sorbitol, 0.035% (v/v) 2-ME, and lyticase (10,000 unit per litter). Cells were incubated at 30°C with shaking at 100 rpm for 1 h and harvested by centrifugation at 1,500*g* for 10 min. The sample was resuspended in homogenization buffer containing 10% (w/v) glycerol, 5 mM Tris-EGTA (pH 7.6), 1.5% (w/v) PVP 40,000, 50 mM Tris-Ascorbate (pH 7.6), 1 mM PMSF, and 0.001% (w/v) Pepstatin A. The cells were broken by sonication on ice at 100 W for 15 min and centrifuged at 530*g* for 20 min at 4°C. The supernatant was collected and then centrifuged at 100,000*g* for 35 min at 4°C. The pellet was dissolved in washing buffer containing 10% (w/v) glycerol, 1 mM Tris-EGTA (pH 7.6), 5 mM Tris-MES (pH 7.5), 1 mM PMSF, and 0.001% (w/v) Pepstatin A and subsequently centrifuged at 100,000*g* for 35 min at 4°C. Pellet was dissolved in 50 mM Tris-MES (pH 7.5) and 10% (w/v) glycerol for further use (Lee et al., 2011).

### Total Protein Extraction From *Arabidopsis*

Roots were grounded in liquid nitrogen and dissolved in protein lysis buffer containing 2% (w/v) SDS, 60 mM Tris-HCl (pH 8.5), 2.5% (w/v) glycerol, 0.2 mM EDTA, 1X (1 tablet/50 ml) protease inhibitor cocktail (Roche), and 1 mM PMSF. Samples were incubated for 10 min at 70°C and then centrifuged at 16,000*g* for 3 min at room temperature. Supernatant was collected and the concentration measured by Dc protein assay kit (Bio-Rad).

### SDS-PAGE and Immunoblot Analysis

Total root proteins and yeast membrane proteins (50 μg) were loaded in each lane for SDS-PAGE followed by electrotransfer onto a PVDF membrane. Each blot was incubated with anti-His (Genetex) or anti-*At*PHT1;1/2/3 antibodies (Huang et al., 2013). Anti-glyceraldehyde-3-phosphate dehydrogenase (Anti-GAPDH) (Genetex) or anti-Actin antibodies (Genetex) were used as the internal controls.

### Bioinformatics Analysis of *At*PHT1;1

The 3D structure of *At*PHT1;1 was predicted with Swiss-model database (https://swissmodel.expasy.org/) using *Pi*PT as a template. All structure figures were prepared with PyMol (https://pymol.org/2/). The topology of *At*PHT1;1 was analyzed by PSIPRED (http://bioinf.cs.ucl.ac.uk/psipred/). The intrinsically disordered (ID) regions of *At*PHT1;1 were predicted with DisEMBL (http://dis.embl.de/) (Linding et al., 2003; Zhao and Xue, 2018). *At*PHT1;1 homologues were searched from NCBI BLAST database. Multiple sequences were aligned using ClustalW (PBIL) (https://npsa-prabi.ibcp.fr). Accession numbers are as follows: *At*PHT1;1 (Q8VYM2) and *At*PHT1;4 (Q96303) (*A. thaliana*), *Sc*PHO84 (P25297) (*S. cerevisiae*), and *Pi*PT (A8N031) (*P. indica*).

## Results

### Structure Prediction of *At*PHT1;1

**Figure 1:**
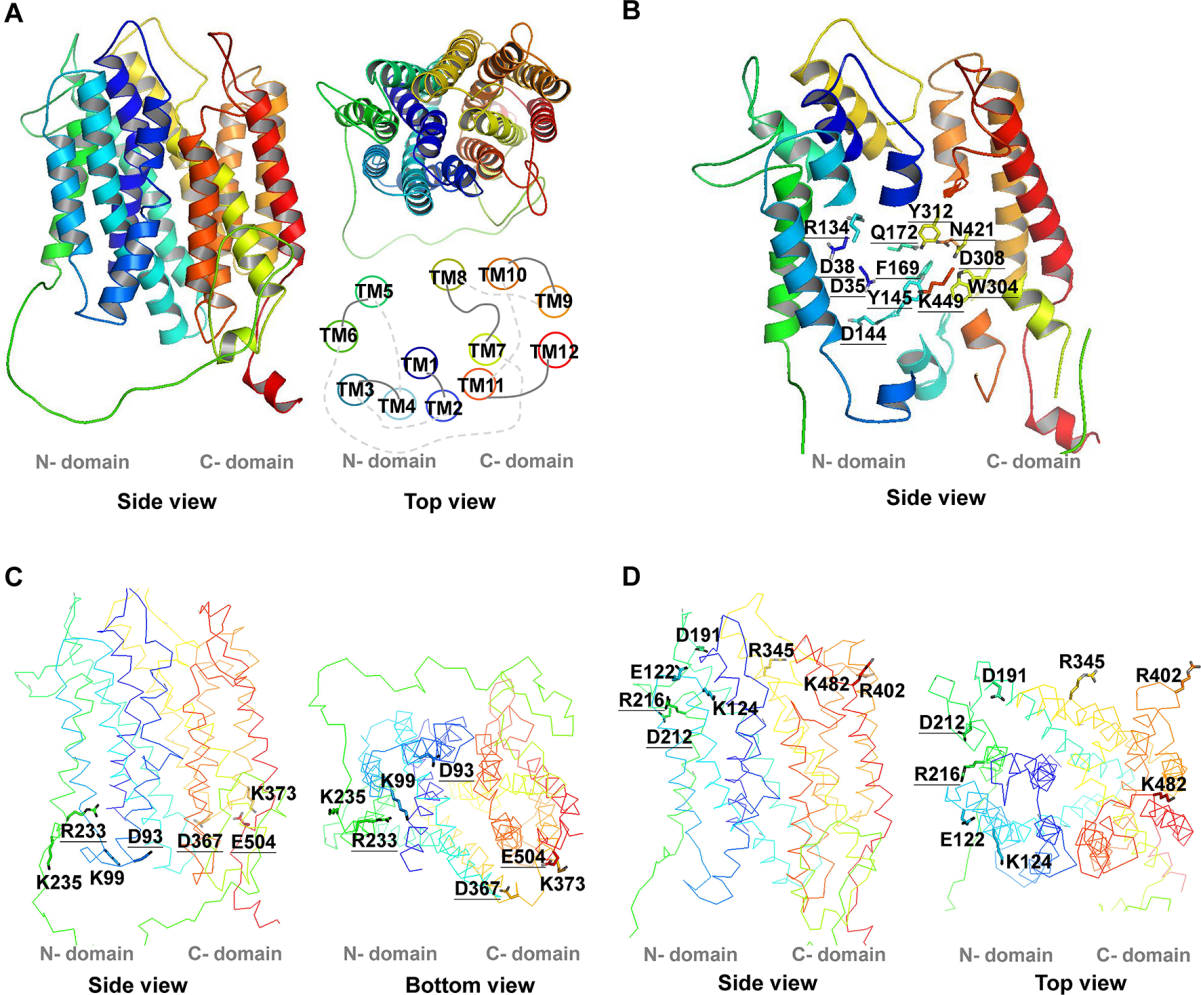
3D structure of *At*PHT1;1. The 3D structure of *At*PHT1;1 was predicted by Swiss-model database using the inward-facing occluded state of *Pi*PT as a template. **(A)** The 3D structure and the TM arrangement. **(B)** Twelve putatively essential residues in the TM. **(C)** Seven charged residues facing the cytoplasmic side (without K16). **(D)** Eight charged residues facing the extracellular side. These structure figures were prepared by PyMol. The underline indicates the conserved amino acid residues among plant and yeast Pi transporters.

**Figure 2:**
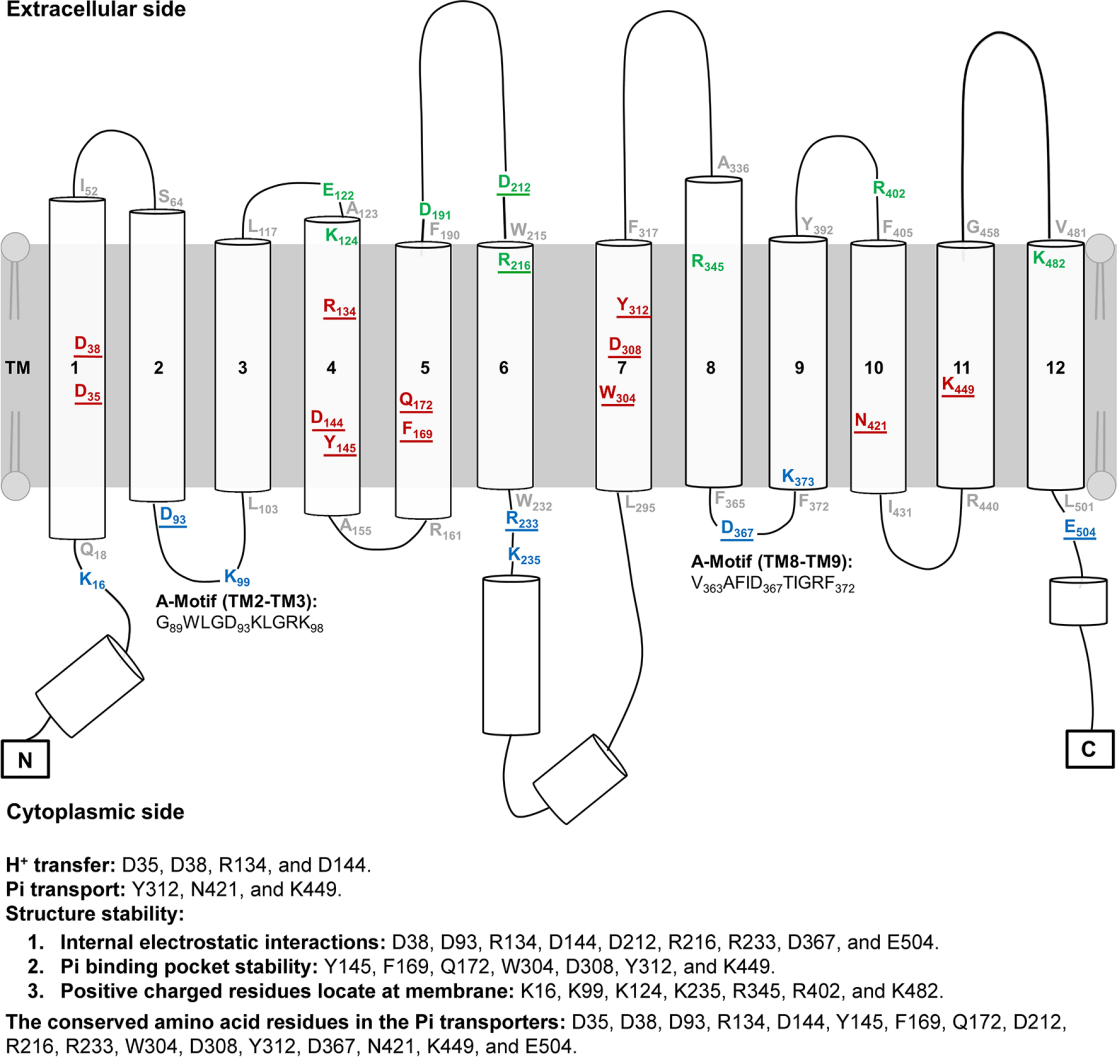
Topology of *At*PHT1;1. The topology of *At*PHT1;1 was predicted according to the protein sequence of *At*PHT1;1 using PSIPRED. Lines indicate loops and coils. Cylinders represent α-Helix. Twenty-eight amino acid residues analyzed in this study are indicated: 8 on the extracellular side (green), 12 in the transmembrane domain (red), and 8 on the cytoplasmic side (blue). The residues located in the bilayer/water interface are shown in gray. Two regions of A-motif are predicted in G_89_WLGD_93_KLGRK_98_ (TM2-TM3) and V_363_AFID_367_TIGRF_372_ (TM8-TM9). The amino acid residues potentially involved in H^+^ transfer, Pi transport and structure stability are indicated. The underline indicates the conserved amino acid residues among plant and yeast Pi transporters.

The secondary structure topology of *At*PHT1;1 was predicted by PSIPRED and its 3D structure simulated by Swiss modeling based on an inward-facing occluded state of *Pi*PT (PDB code: 4J05) (**Figures 1** and **2**; Pedersen et al., 2013). Three conformational states similar to most MFS members, an outward open state, an inward open occluded, and a ligand free occluded state, were also suggested for *At*PHT1;1 (Sun et al., 2012; Jiang et al., 2013; Zheng et al., 2013). *At*PHT1;1 consists of 12 TMs, which are divided into two domains linked by a large flexible loop, with N- and C-termini facing the cytosolic side (**Figures 1** and **2**). Two conserved sequences of the so-called A-motif were found as well in TM2-TM3 (corresponding to G_89_WLGD_93_KLGRK_98_ in *At*PHT1;1) and TM8-TM9 (corresponding to V_363_AFID_367_TIGRF_372_ in *At*PHT1;1) loops, respectively (**Figure 2** and **Figure S1**; Jiang et al., 2013; Quistgaard et al., 2016). Residues in the A-motif are believed to form various salt bridges and internal gates, providing a force for TM movement to modulate different conformational states of MFS (**Table S3**; Jiang et al., 2013; Quistgaard et al., 2016). Numerous putative intracellular hydrogen bonds were also proposed to stabilize the structure and/or substrate interactions (**Figure S2**; Kumar and Nussinov, 2002). In addition, several intrinsically disordered (ID) regions of *At*PHT1;1 were identified in loops and coils (the blue line in **Figure S3**), whose coordinates are usually missing in the X-ray crystal structure (Lin et al., 2012). The ID regions are composed of skewed amino acids with a low content of bulky hydrophobic amino acids (V, L, I, M, F, W, and Y) and a high proportion of polar and charged amino acids (N, S, P, E, K, and on occasion, G and A), comprising more flexible and diverse structural ensembles (Dyson and Wright, 2005; Oldfield and Dunker, 2014; Tusnády et al., 2015).

### Selection of Potential Key Amino Acid Residues Involved in the Pi Transport Activity of *At*PHT1;1

To identify the amino acid residues important for H^+^-coupled Pi transport activity of *At*PHT1;1, we selected 27 hydrophilic residues and one hydrophobic residue (F169) located in the TM helix or bilayer/water interface for analysis (**Figure 2**). Hydrophilic residues are able to exert non-covalent interactions with substrates, possibly involved in structural stability, H^+^ transfer, Pi transport/binding, or the electrostatic interaction during conformational changes. Among them, six charged residues (D35, D38, R134, D144, D308, and K449), which are highly conserved in PHS (**Figure S1** and **Table S2**), are proposed to participate in H^+^ and Pi transport because of positioning in the central pathway of the 3D structure (**Figure 1B**; Samyn et al., 2012; Pedersen et al., 2013). An additional 16 charged residues located in the bilayer/water interface (**Figure 2**) are hypothesized to be involved in substrate uptake or release (Jiang et al., 2013; Pedersen et al., 2013; Quistgaard et al., 2016). Besides charged residues, six neutral residues (Y145, F169, Q172, W304, Y312, and N421) corresponding to the amino acids of *Pi*PT for Pi binding were also included in the analysis (Pedersen et al., 2013).

These 28 residues were then classified into three groups based on their predicted location at the TM helix, extracellular side, or cytoplasmic side, respectively (**Figures 1** and **2**). To characterize their contribution to Pi transport activity, they were substituted by alanine individually for functional analyses in yeast and *Arabidopsis* mutants.

### Functional Analysis of *At*PHT1;1 in Yeast *pam2* Mutant

**Figure 3:**
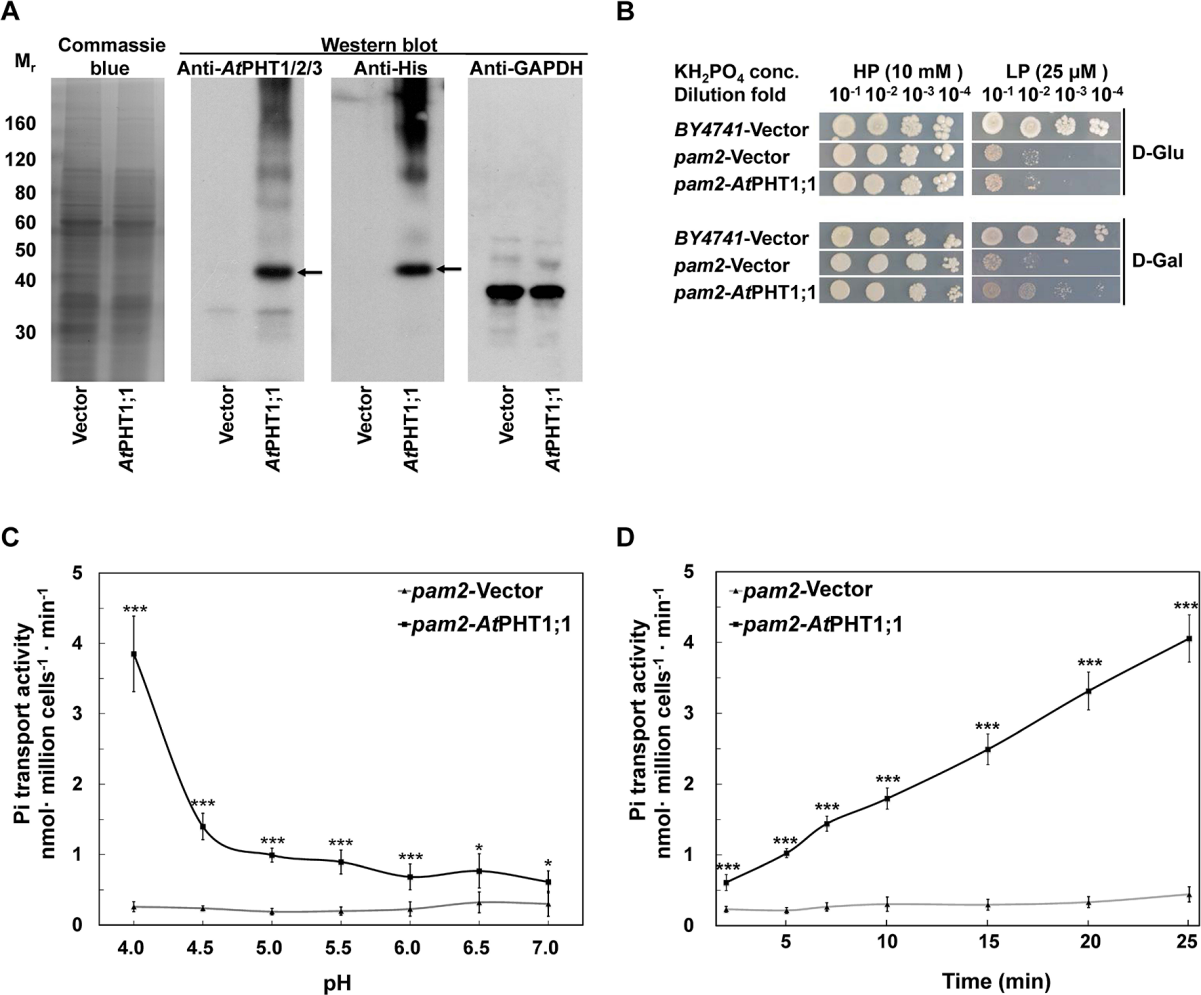
Characterization of *At*PHT1;1 expression and function in *S. cerevisiae*. **(A)** Immunoblot analysis of protein expression. Left panel: Coomassie Blue staining. Middle panels: *At*PHT1;1 proteins detected by anti-*At*PHT1;1/2/3 or Anti-His antibody. Right panel: glyceraldehyde-3-phosphate dehydrogenase (GAPDH) as a control. **(B)** Complementation growth analysis of *pam2* yeast expressing *At*PHT1;1. **(C)** pH dependence of Pi transport. **(D)** Time course of Pi transport at pH 4.0. Error bars represent _SD_ (n = 3). Results were reproducible in at least two independent experiments. Asterisks indicate a stastically significant difference from the vector control. *P<0.05 and ***P<0.001, student’s t-test.

The full-length coding sequence of *At*PHT1;1 was fused with His_6_-tag at the C terminus and constructed into pYES2 yeast expression vector, in which the expression of *At*PHT1;1 is repressed by glucose but induced by galactose. For functional complementation analysis, the resulting constructs were individually transformed into *S. cerevisiae* strain *pam2* the activities of both high-affinity Pi transporters (*PHO84* and *PHO89*) of which were lost (Martinez and Persson, 1998). The expression of WT *At*PHT1;1 was first examined by immunoblotting using antibodies directly against *At*PHT1;1 or poly-His (**Figure 3A**). Although the molecular mass of *At*PHT1;1 was predicted to be 58 kDa, it ran at 40 kDa on SDS-PAGE (Liu et al., 2011). *At*PHT1;1 proteins were detected by both antibodies in galactose-grown *pam2* cells transformed with *At*PHT1;1 (*pam2*-*At*PHT1;1) but not in those having the empty vector control (*pam2*-vector), indicating successful expression of *At*PHT1;1 in the yeast (**Figure 3A**).

To examine whether the expression of *At*PHT1;1 is able to complement the growth of *pam2*, the yeast cells of *pam2*-vector and *pam2*-*At*PHT1;1 were diluted serially from 10^−1^ to 10^−4^ and spotted on high Pi (HPi, 10 mM KH_2_PO_4_) or low Pi (LPi, 25 μM KH_2_PO_4_) agar media supplemented with glucose or galactose as the carbon source (**Figure 3B**). The growth on the plate supplemented with glucose was taken as a background control. BY4741 WT yeast strain containing all functional five Pi transporters (two high affinities and three low affinities) was included as a positive control (Jensen et al., 2003). Under HPi conditions, all the yeast cells grew well (**Figure 3B**). However, under LPi conditions, *pam2*-vector showed very limited growth no matter where it was supplemented with glucose or galactose (**Figure 3B**). Growth complementation was observed when *pam2*-*At*PHT1;1 was grown under LPi media supplemented with galactose but not glucose (**Figure 3B**). These results demonstrate the successful expression of functional *At*PHT1;1 in yeast and also suggest that *At*PHT1;1 is a high-affinity Pi transporter.

We next measured the ^33^Pi uptake activities of *pam2*-vector and *pam2*-*At*PHT1;1 grown under a low Pi concentration (100 μM KH_2_PO_4_). A similar Pi concentration was used in previous studies on *Sc*PHO84 (Samyn et al., 2012; Samyn et al., 2016). To examine the pH dependence, we determined the transport activity of *pam2*-*At*PHT1;1 in the solution with different pH (pH 4.0 to 7.0) for 20 min and found its activity increased concomitantly with the decrease of pH (**Figure 3C**), suggesting the coupling of H^+^ with Pi transport. Furthermore, *pam2*-*At*PHT1;1 showed a time-dependent Pi transport activity within a 25-min period of incubation (**Figure 3D**). As a negative control, *pam2*-vector displayed very low activity under all conditions tested, indicating that the Pi transport activity detected is specifically contributed by *At*PHT1;1. Based on these observations, the subsequent transport assay in yeast was conducted at pH 4.0 and measured after 20-min incubation.

### Functional Analysis of *At*PHT1;1 in *Arabidopsis* Mutant

**Figure 4:**
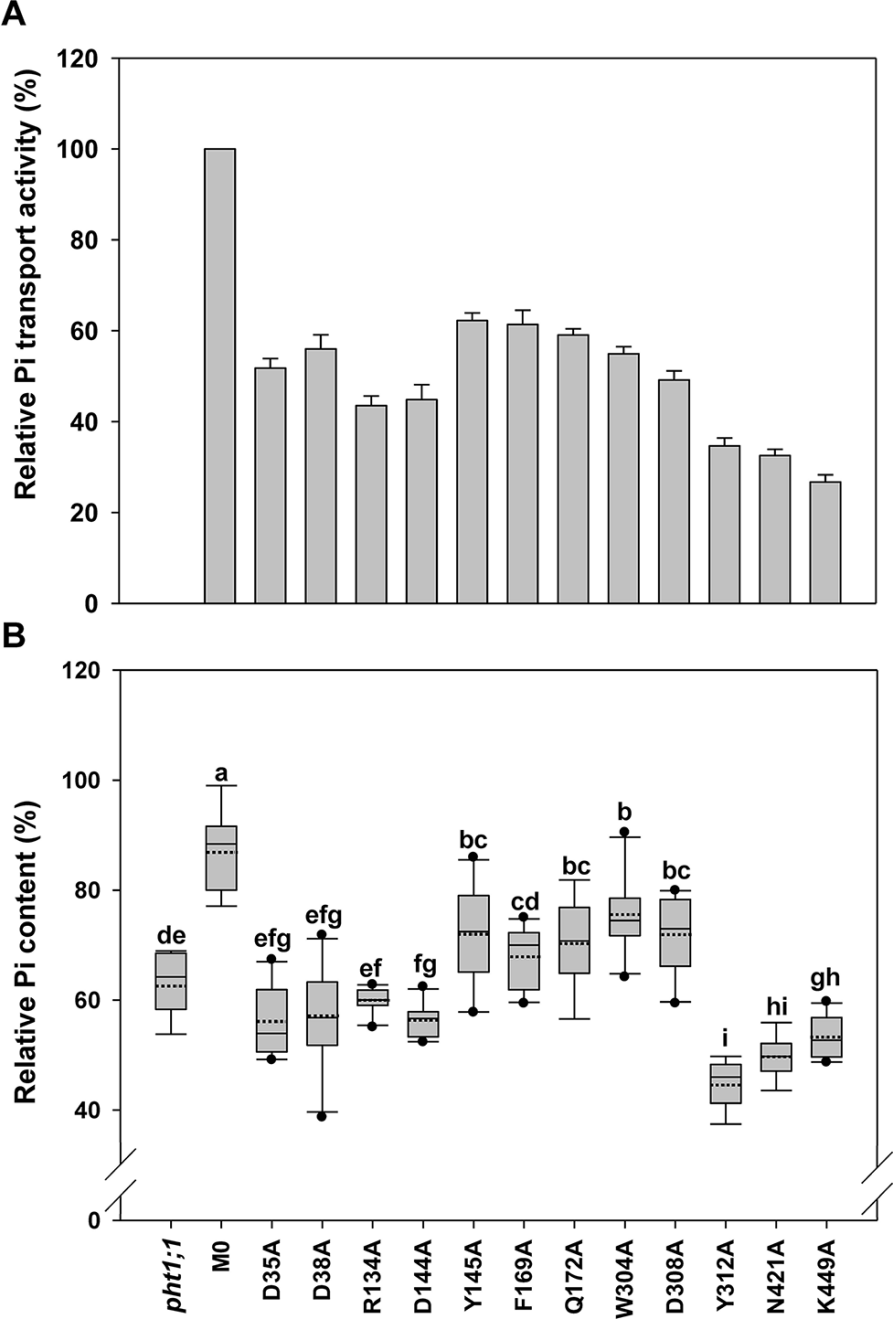
Evaluation of transmembrane-localized amino acid residues of *At*PHT1;1 by functional analyses in yeast and *Arabidopsis*. **(A)** Relative Pi transport activity in yeast. The activity of each variant was normalized with respective *At*PHT1;1 protein amount in **Figure S5** and presented as the percentage of WT *At*PHT1;1 expression in *pam2*. Error bars represent _SE_ (n = 3). Results were reproducible in at least two independent experiments. **(B)** The relative Pi content of transgenic seedlings is indicated as a percentage of WT *Arabidopsis*. Data are shown as box plots (n = 8-10 independent T2 lines). The boundaries of the boxes indicate the 25th and 75th percentiles. The mean and median are marked by dashed and black lines within the box, respectively. Error bars above and below the box indicate the 90th and 10th percentiles. Value outside the 10th and 90th percentiles is displayed as a single dot. Different low ease letters represent a significant difference among lines. (ANOVA, *P* < 0.05).

**Figure 5:**
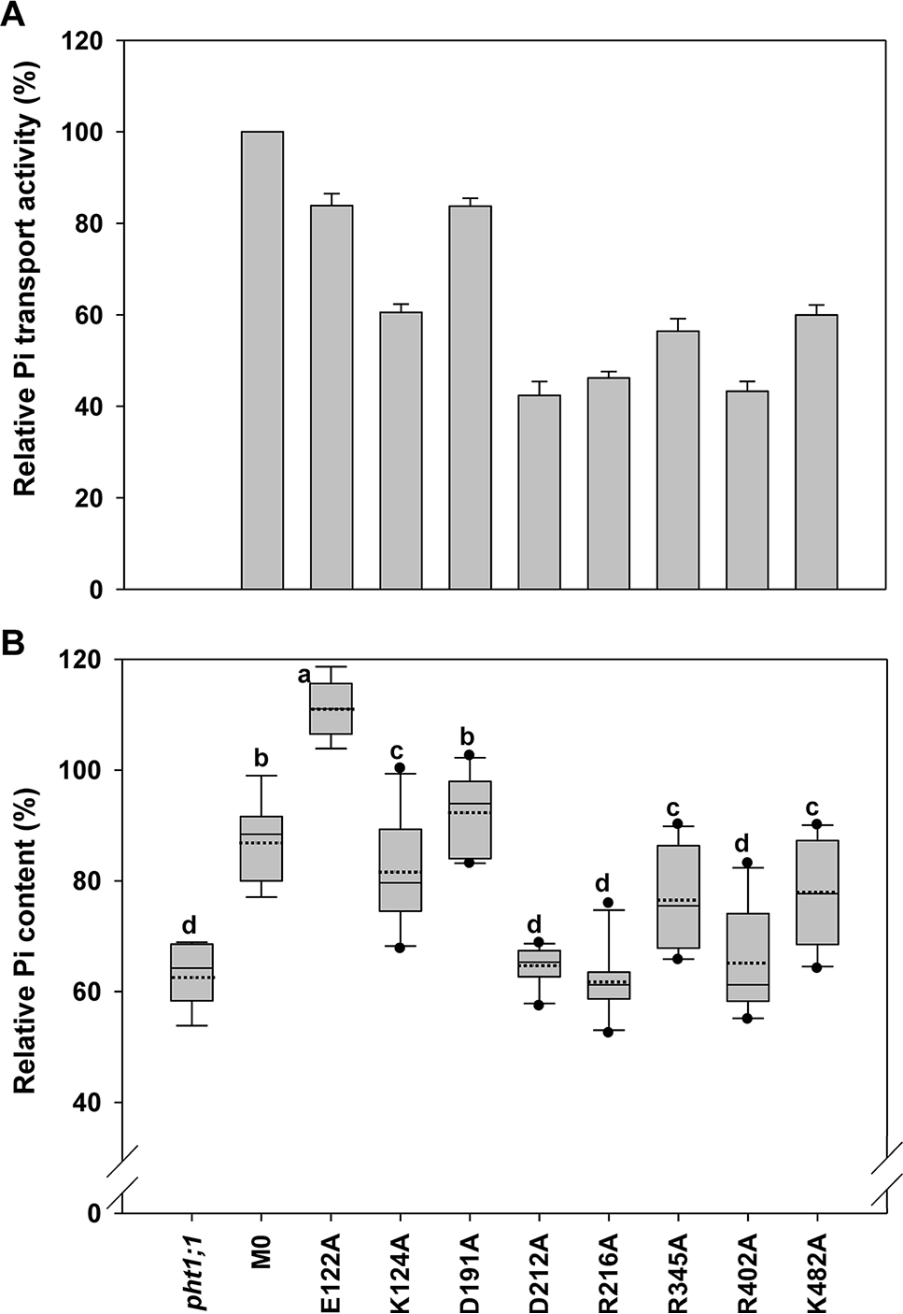
Evaluation of amino acid residues on the extracellular side of *At*PHT1;1 by functional analyses in yeast and *Arabidopsis*. **(A)** Relative Pi transport activity in yeast. The activity of each variant was normalized with respective *At*PHT1;1 protein amount in **Figure S5** and presented as the percentage of WT *At*PHT1;1 expression in *pam2*. Error bars represent _SE_ (n = 3). Results were reproducible in at least two independent experiments. **(B)** The relative Pi content of transgenic seedlings is indicated as the percentage of WT *Arabidopsis*. Data are shown as box plots as described in **Figure 4** (n = 8-10 independent T2 lines). Different low case letters represented a significant difference among lines. (ANOVA, *P* < 0.05).

**Figure 6:**
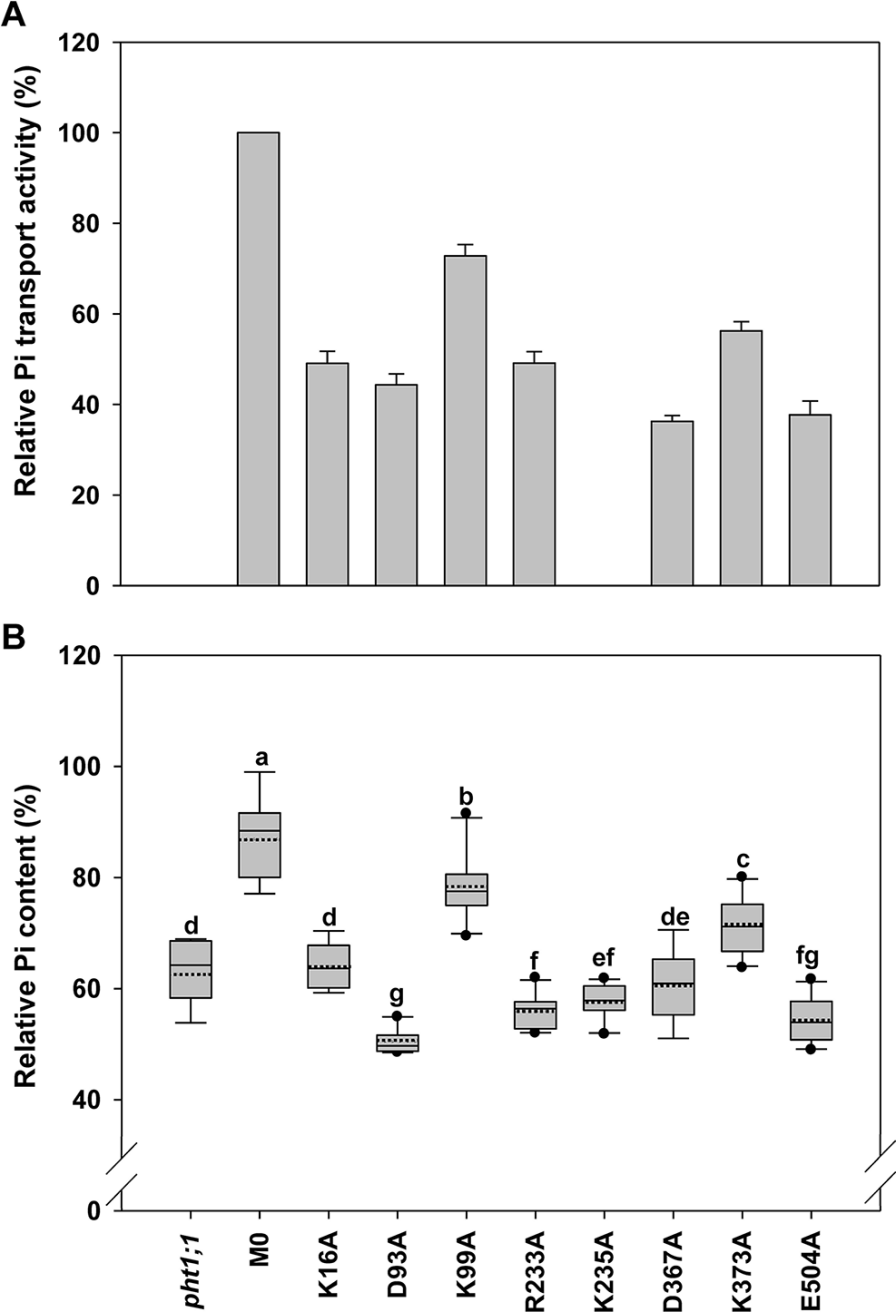
Evaluation of amino acid residues on the cytoplasmic side of *At*PHT1;1 by functional analyses in yeast and *Arabidopsis*. **(A)** Relative Pi transport activity in yeast. The activity of each variant was normalized with respective *At*PHT1;1 protein amount in **Figure S5** and presented as the percentage of WT *At*PHT1;1 expression in *pam2*. Error bars represent _SE_ (n = 3). Results were reproducible in at least two independent experiments. **(B)** The relative Pi content of transgenic seedlings is indicated as the percentage of WT *Arabidopsis*. Data are shown as box plots as described in **Figure 4** (n = 8-10 independent T2 lines). Different low case letters represent a significant difference among lines. (ANOVA, *P* < 0.05).

It was previously demonstrated that loss-of-function of *pht1;1 Arabidopsis* mutants accumulated less Pi than WT plants as a result of reduced Pi uptake activity when grown under Pi replete conditions (Shin et al., 2004). To identify an optimal condition for evaluating the complementation, the Pi content of *Arabidopsis* WT and *pht1;1* seedlings grown in the medium containing different Pi concentrations (from 25 to 1000 μM) was measured. Consistent with the previous findings, *pht1;1* showed significantly lower Pi content than the WT when the Pi concentration was above 250 μM (**Figure S4**). The concentration of 1000 μM Pi was chosen for the following complementation analysis because the reduction of Pi content in *pht1;1* (∼60% of WT) was most significant under this condition (**Figure S4**). When the WT *AtPHT1;1* was transformed into *pht1;1* seedlings under the control of its native promoter (designated as M0), the Pi content was restored to 87 ± 7% of WT (**Figures 4**–**6**). M0 was therefore used as a complementation control. For each mutant variant, the Pi content of 8 to 10 independent T2 lines were measured and illustrated by box plots. The original and relative Pi contents of individual lines were shown in **Supplementary Material (Data S1)**.

### TM-Localized Amino Acid Residues of *At*PHT1;1 Crucial for Pi Transport

The first group comprises 12 residues (D35, D38, R134, D144, Y145, F169, Q172, W304, D308, Y312, N421, and K449) located within the TM helix, which are highly conserved in PHS (**Figure S1** and **Table S2**). Based on the structure model of *Pi*PT proposed previously, D35 (TM1), D38 (TM1), R134 (TM4), and D144 (TM4) are suggested to interact with H^+^. The rest, Y145 (TM4), F169 (TM5), Q172 (TM5), W304 (TM7), D308 (TM7), Y312 (TM7), N421 (TM10), and K449 (TM11), are believed to reside in the putative Pi binding pocket (**Figure 2** and **Table S2**; Samyn et al., 2012; Pedersen et al., 2013; Samyn et al., 2016). It is also believed that the residues in core helices of TMs 1, 4, 7, and 10 at the central path of MFS members possibly engage in substrate translocation, while those in the middle helices of TMs 2, 5, 8, and 11 participate in substrate binding and co-transport coupling (Yan, 2015). Likewise, formation of several hydrogen bonds were predicted among D38, R134, D144, Q172, W304, D308, Y312, and K449 in the inward-facing occluded state of *At*PHT1;1 (**Figures S2A–C, E, H, I, K**).

When these 12 mutated variants were expressed in *pam2* yeast, all of them were expressed well (**Figure S5**). However, they showed partial complementation to various extents, from 25% to 60% of *pam2*-*At*PHT1;1 activity (**Figure 4A**). Upon the assessment of Pi content in the plant system, D35A, D38A, R134A, and D144A variants displayed no complementation; but Y145A, F169A, Q172A, W304A, and D308A variants exhibited partial complementation (**Figure 4B**). Surprisingly, Y312A, N421A, and K449A variants did not complement *pht1;1* but provoked further reduction of the Pi content (**Figure 4B**). The proteins of these 12 variants were all expressed *in planta* (equivalent to or higher than the level of WT or M0) (**Figure S6**). These results demonstrate that D35, D38, R134, D144, Y312, N421, and K449 are essential residues for the activity of *At*PHT1;1 *in planta*; however, Y145, F169, Q172, W304, and D308 also play a role in Pi transport activity (**Figure 4**).

### Key Amino Acid Residues of *At*PHT1;1 on the Extracellular Side

Eight amino acid residues of *At*PHT1;1, E122 (TM3-TM4), K124 (TM4), D191 and D212 (TM5-TM6), R216 (TM6), R345 (TM8), R402 (TM9-TM10), and K482 (TM12), predicted to face the extracellular side, were selected for functional studies (**Figure 2** and **Figure S2**). Since the TMs 3, 6, 9, and 12 of MFS members were proposed to play a role in maintaining structural integrity (Yan, 2015), we postulated that the residues located at these TMs, E122, D191, D212, R216, R402, and K482, may participate in structural stability. Furthermore, our bioinformatics analysis revealed that E122, D191, D212, R402, and K482 are located at the ID regions of *At*PHT1;1 (**Figure S3**) and E122, D212, R216, and R345 are capable of forming hydrogen bonds (**Figures S2A, B, D, F, G**), of which D212 and R216 are highly conserved in PHS (**Figure S1**).

When the Pi transport activity of these eight variants was assessed in yeast, D212A R216A, and R402A variants showed only 40% of *pam2*-*At*PHT1;1 activity; K124A, R345A, and K482A variants retained 60% of the activity; however, E122A and D191A variants still kept 83% of the activity (**Figure 5A**). The protein of these eight variants could be detected in the yeast (**Figure S5**) and the plant cells as well (**Figure S6**). In the *pht1;1* mutant, D212A, R216A, and R402A variants showed no complementation, while K124A, R345A, and K482A variants displayed partial complementation (**Figure 5B**). On the other hand, D191A variant could fully complement the *pht1;1* mutant with Pi content similar to the M0 control (**Figure 5B**). Remarkably, E122A variant accumulated higher Pi than the M0 control and WT plants, suggesting that replacement of E122 with alanine enhances the Pi transport activity of *At*PHT1;1 (**Figure 5B**). Because E122 resides in the ID regions (**Figure S3**), we surmise that the small side chain of alanine may reduce the internal interaction, improving structure ensembles and adaptability for the enhancement of activity. In general, the outcomes of complementation from yeast and plant systems are in a good agreement. Our results suggest that D212, R216, and R402 are essential for the activity of *At*PHT1;1, whereas K124, R345, and K482 have relatively minor roles. Nonetheless, changes of E122 and D191 to alanine, respectively, showed no impairment of the activity.

### Key Amino Acid Residues of *At*PHT1;1 on the Cytoplasmic Side

Eight amino acid residues on the cytoplasm side of *At*PHT1;1, i.e., K16 (N terminus), D93 and K99 (TM2-TM3), R233 and K235 (TM6-TM7), D367 (TM8-TM9), K373 (TM9), and E504 (C terminus) (**Figure 2**), were selected for investigation. D93, R233, D367, and E504 are highly conserved in PHS (**Figure S1**), E504 is located in ID regions (**Figure S3**), and R233, D367, K373, and E504 were predicted to form hydrogen bonds (**Figures S2A, B, E, J, L**). The proteins of these variants could be detected in the yeast cells except K235A which was then not included for subsequent assessment (**Figure S5**). Five of the variants (K16A, D93A, R233A, D367A, and E504A) showed 40% to 50% of *pam2*-*At*PHT1;1 activity, while K99A and K373A variants displayed 60% to 70% of activity (Figure 6A). In the plant system, all eight variants were expressed well (**Figure S6**). Apart from K99A and K373A which produced partial complementation, the rest (K16A, D93A, R233A, K235A, D367A, and E504A) failed to complement *pht1;1* mutants (**Figure 6B**). Of note, D93A, R233A, K235A, and E504A variants had an even lower Pi level than *pht1;1* mutants (**Figure 6**). The results of *in planta* analysis suggest essential roles of K16, D93, R233, K235, D367, and E504, but relatively minor roles of K99 and K373 for the Pi transport activity of *At*PHT1;1.

## Discussion

To sustain plant growth and development, Pi is acquired by the roots and subsequently transported and allocated to different tissues by the plasma membrane-bound PHT1 Pi transporters belonging to MFS. Crystal structure analyses of several MFS members have established various models revealing their transport mechanisms as consecutive conformational states modulated by internal salt bridges, hydrogen bonds, and substrate interactions (Jiang et al., 2013; Yan, 2015; Quistgaard et al., 2016; Ke et al., 2017). However, the specific amino acid residues involved in each step of the Pi transport process are still not well defined, and particularly lack experimental validations. The *At*PHT1;1, a key player for initial Pi uptake at the rhizosphere, is identified as a H^+^/Pi co-transporter predominantly expressed at the plasma membranes of root cells (Nussaume et al., 2011; Johri et al., 2015). In the present study, we employed various computational structure analyses followed by experimental validation of functional complementation in yeast and *Arabidopsis* mutants to elucidate the Pi transport mechanism of *At*PHT1;1. Although the overall results from yeast and *Arabidopsis* showed a similar trend, the effect of complementation among different variants in yeast is less discernible than in *Arabidopsis*. We speculate that the discrepancy between yeast and plant complementation results may be attributed to the different promoters driving the expression of *At*PHT1;1. One is a galactose-induced strong yeast promoter, the other is an endogenous native promoter of PHT1;1 with spatial and temporal regulation in plants. Furthermore, the different nature of these two organisms should be taken into account.

In addition to the abundance of expressed *At*PHT1;1 protein (**Figure S6**), its proper targeting to plasma membranes is crucial for determining the transport activity. We have selected 11 variants (3-4 variants from each category of location, D35A, D93A, D144A, D212A, R216A, R233A, R345A, D367A, K373A, R402A, and K449A), which showed no or partial complementation in *Arabidopsis* and examined their localization by YFP tagging together with M0 control. No change of their plasma membrane targeting was observed (**Figure S7**), indicating that the reduced activity in these variants is not caused by mis-localization. The following discussion is made mainly based on the results in *Arabidopsis*. The structure–function roles of several amino acid residues in *At*PHT1;1 are explicated according to their location across the membrane.

### Essential Residues Within TM Domain

#### Essential Residues for Pi Transport

The Y145, F169, Q172, W304, D308, Y312, N421, and K449 residues conserved in the PHS family were considered to form a putative Pi binding pocket implicated from the *Pi*PT 3D structure (**Figure S1** and **Table S2**; Pedersen et al., 2013). The structure prediction suggests that Q172, D308, Y312, and N421 residues are located near the extracellular side, whereas Y145, F169, W304, and K449 residues are close to the cytoplasmic side (**Figure 1B**). Our complementation analysis showed that Y145A, F169A, Q172A, W304A, or D308A variants impair the Pi transport activity and Y312A, N421A, or K449A variants totally abolish activity of *At*PHT1;1 and even reduce Pi basal level of WT (**Figure 4B**). We proposed that Y312 and N421 residues might interact with Pi initially near the extracellular side and then deliver Pi to the Pi binding pocket. Additionally, the aromatic moieties of the Y145 and F169 residues (**Figure 1B**) and the electrostatic interactions derived from the hydrogen bonds within the residues of Y312-Q172-Y359-N417-T355, W304-D308, and K449-T81 located in the Pi binding pocket (**Figures S2A, B, H, I, K**) could stabilize the Pi binding. The conserved residue of D358 in *Sc*PHO84 and D324 in *Pi*PT (corresponding to D308 in *At*PHT1;1) were suggested to participate directly in Pi transport by protonation/deprotonation to trigger off conformational changes (**Table S2**; Samyn et al., 2012; Pedersen et al., 2013; Samyn et al., 2016). In our analysis, however, the D308A variant still retained partial activity (**Figure 4B**) and the D308 residue could form electrostatic interaction with the W304 residue (**Figure S2H**). Therefore, D308 residue may not be directly involved in Pi transport; instead, the electrostatic interaction between D308 and W304 residues may stabilize the structure to allow the access of Pi to the binding pocket by reducing the repulsive force. Formation of electrostatic interaction between the positively charged moiety of K449 and Pi presumably facilitates Pi release to the cytoplasmic side, which is essential for the activity of *At*PHT1;1. However, mutation of its corresponding residues in *Sc*PHO84 (K492A, K492E, and K492Q) still maintained the activity in spite of reduction of affinity to Pi (**Table S2**; Samyn et al., 2012). The discrepancy between these two homologues in different organisms is currently unknown and requires further examination.

#### Essential Residues for H^+^ Transfer

The R134, D38, D35, and D144 residues conserved in the PHS family are aligned in turn from the extracellular to cytoplasmic sides (**Figures 1** and **2**), possibly comprising a H^+^ transport channel. The negative charges of D38, D35, and D144 (corresponding to D48, D45, and D149 in *Pi*PT, respectively) form a consecutive pathway route to facilitate the H^+^ across membranes (Pedersen et al., 2013). Mutation in any of them into alanine failed to restore the Pi level in *pht1;1* mutants (**Figure 4B**). The electrostatic interaction between D38 and R134 residues, as proposed from analogous interactions in different 3D structure states of XylE (D27/R133 in XylE) and GLUT1 (N29/R126 in GLUT1) would presumably accelerate H^+^ transfer to D35 and D144 residues for final release (Wisedchaisri et al., 2014; Yan, 2015; Ke et al., 2017). In yeast, mutation of R168 or D178 residue of *Sc*PHO84 (corresponding to R134 or D144 residue in *At*PHT1;1; **Table S2**) also resulted in significant reduction in Pi uptake activity (Samyn et al., 2012). Taken together, D35, D38, R134, and D144 residues presumably engaged in H^+^ transfer are indispensable for the activity of *At*PHT1;1, demonstrating the coupled of H^+^ to Pi transport.

### Essential Residues at Bilayer Interfaces on Extracellular or Cytoplasmic Sides

#### Essential Residues for Conformational Changes, Internal Electrostatic Interactions, and Structural Stability

Our functional assay revealed that several charged residues on the extracellular or cytoplasmic sides, such as D93, D212, R216, R233, D367, K373, and E504 residues, are essential for the activity of *At*PHT1;1 (**Figures 5** and **6**). According to the predicted 3D structure of *At*PHT1;1, D93 and D367 residues located in the A-motif might form serial electrostatic interactions among the surrounding residues, such as D93-R97-E154-R437 and D367-R371-E430-R161-A163-K373-E504-F372 (**Figure S2** and **Table S3**; Sun et al., 2012; Jiang et al., 2013; Pedersen et al., 2013; Wisedchaisri et al., 2014; Quistgaard et al., 2016). In MFS, such interactions are proposed to trigger TM movement during conformational changes followed by the substrate uptake and release (Sun et al., 2012; Jiang et al., 2013; Pedersen et al., 2013; Yan, 2015; Quistgaard et al., 2016). D93A and D367A variants totally abolished the activity of *At*PHT1;1 (**Figure 6B**). We thus propose that the conformational changes mediated by D93 and D367 residues are indispensably required for Pi transport. Additional electrostatic interactions provided by D212, R216, R233, and the A-motif residues might also play a role in maintaining the internal structure stability of *At*PHT1;1.

#### Residues for Other Functionalities

**Figure 7:**
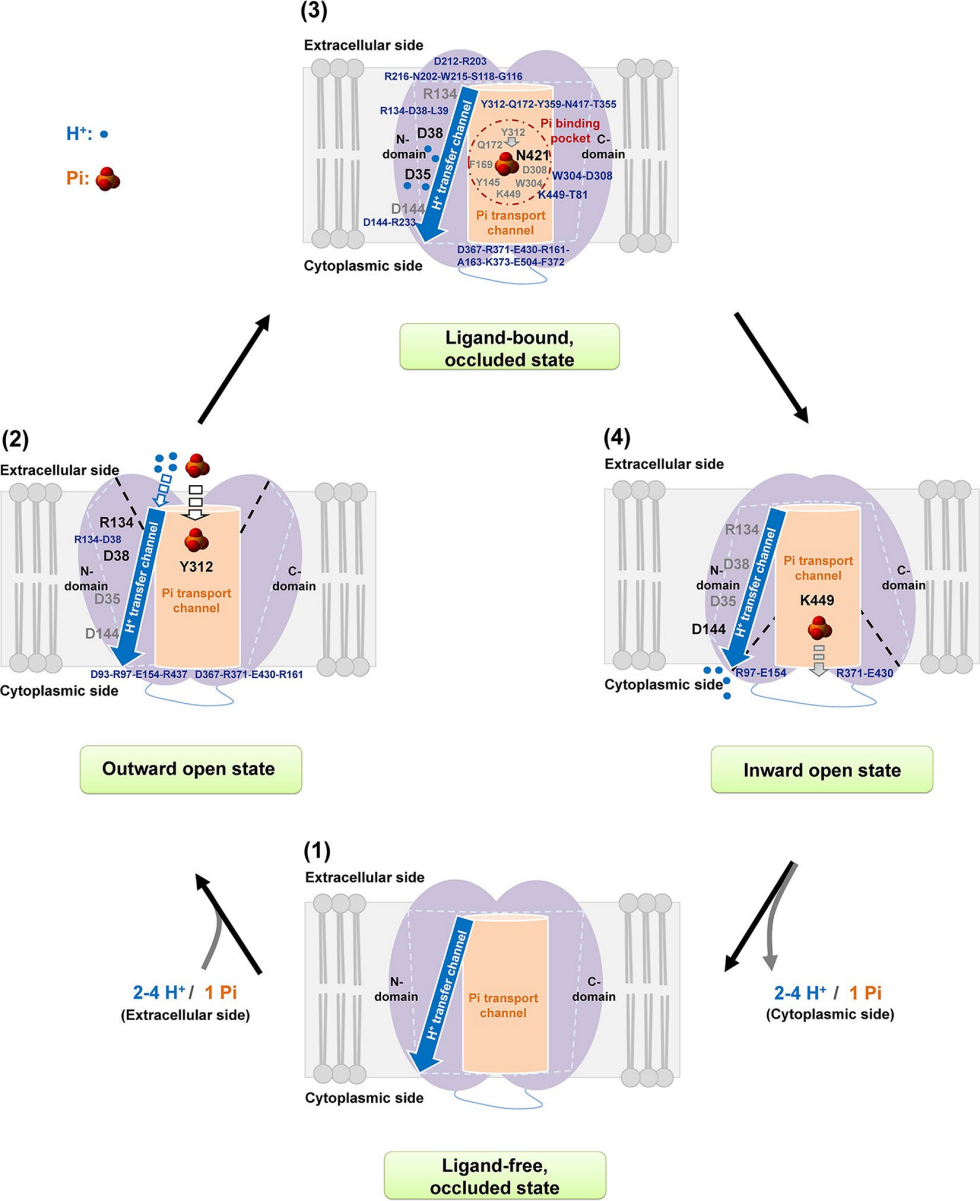
Proposed mechanism of Pi transport of *At*PHT1;1. A working scheme is proposed for H^+^/Pi transfer in *At*PHT1;1 comprising subsequently initial ligand-free occluded **(1)**, outward open **(2)**, inward facing occluded **(3)**, and finally inward open **(4)** states. See text for the details.

Among the 28 residues examined, only E122A and D191A variants could fully restore Pi to the level of WT *At*PHT1;1 transgenic line (M0) or even higher (**Figure 5B**). E122 and D191 residues are predicted in ID regions (**Figure S3**), which provide flexible and diverse structural ensembles (Dyson and Wright, 2005; Oldfield and Dunker, 2014; Tusnády et al., 2015). Replacement of E122 and D191 residues with alanine, which contains a small and non-charged side chain, may reduce internal electrostatic interactions for structure flexibility. Increased Pi uptake activity, as seen in the case of E122A variant, offers a future opportunity to engineer crops with improved Pi acquisition efficiency. Furthermore, mutations in several positively charged residues on the bilayer/water interface (e.g., K16A, K99A, K124A, R345A, K373A, R402A, and K482A) showed a moderate impact on its function (**Figures 6B** and **7B**). We hypothesize that these positively charged residues might provide structural flexibility for stabilizing interactions with negatively charged head groups of the lipid molecules in transmembrane proteins (Tusnády et al., 2015).

It is interesting to note that several variants (e.g., D93A, D144A, R233A, K235A, Y312A, N421A, K449A, and E504A) display dominantly negative effects. The mutation not only failed to complement but even further reduced basal Pi content of *pht1;1* mutants (**Figures 4B** and **6B**). Because several of them (D93A, D144A, R233A, and K449A) target to the plasma membranes properly (**Figure S7**), we thus hypothesize that these amino acid residues might participate in the reorganization of functional subunit–subunit interactions. In the 3D structure, the interface residues in many transmembrane proteins are likely to participate in subunit or complex interactions *via* electrostatic interactions of hydrophobic residues, hydrogen bonds, and salt bridges (Lin et al., 2012). As a matter of fact, previous *in vivo* and *in vitro* interaction assays showed that PHT1;1 and PHT1;4 are able to form homomeric or heteromeric oligomers, and their oligomerization may offer a means to regulate transporter activity (Fontenot et al., 2015). Therefore, the dominantly negative effect as seen in these variants might result from the alteration of heteromeric oligomers with PHT1;4 or other PHT1 members and eventually impair Pi accumulation.

### A Mechanistic Model of Pi Transport of *At*PHT1;1

Based on the current four-state model of initial ligand-free occluded, followed by outward open, ligand-bound occluded, and finally inward open states (Yan, 2015), a Pi transport mechanism of *At*PHT1;1 is proposed as shown in **Figure 7**. When Pi approaches, it interacts with Y312 residue first and electrostatic interactions within D38-R134, D93-R97-E154-R437, and D367-R371-E430-R161 are provoked into an outward open state. In the substrate bound occluded state, Pi is translocated to N421 in the Pi binding pocket where two aromatic moieties of Y145 and F169 residues and the electrostatic interactions within the Y312-Q172-Y359-N417-T355 region and within W304-D308, and K449-T81 pairs, support structural stability for Pi binding. Concurrently, the amine side chain of the R134 residue might bind carboxylic main chains of D38 and L39 residues, allowing the side chain of D38 residue to transfer H^+^ to D35 residue. Subsequently, the hydrogen bonds within D144-R233, D212-R203 pairs and among R216-N202-W215-S118-G116 and D367-R371-E430-R161-A163-K373-E504-F372 regions would provide the state transition in the substrate bound occluded state. Eventually, H^+^ is released by D144 residue while Pi interacts with K449 residue for its later release and two salt bridges of R97-E154 and R371-E430 pairs would maintain state transition to a ligand-free occluded state.

## Conclusions

Based on bioinformatic prediction followed by experimental validation, a legitimate mechanistic model is proposed to elucidate the transport of a plant H^+^-coupled Pi transporter. The amino acid residues crucial for Pi binding, H^+^ transport, conformational states, and structural stability were unveiled. P is a non-renewable resource and has low availability in many soils. Results of this study not only provide fundamental knowledge of the role of amino acid residues during the course of Pi transport across membranes in plants but also offer a basis for future genetic engineering or gene editing of Pi acquisition in crops to achieve sustainable agriculture.

## Data Availability

All datasets generated for this study are included in the manuscript/**Supplementary Files**.

## Author Contributions

Y-YL and J-LL performed the experiments. Y-YL, R-LP, and T-JC wrote the manuscript. All authors have participated in experimental design the data analysis through the course of this study. All authors have read and approved the manusript.

## Funding

This work was supported by grants from Ministry of Science and Technology, Taiwan, Republic of China to R-LP (MOST 103-2311-B-007-001-MY2, MOST 104-2321-B-007-002, MOST 105-2311-B-007-011-MY2), and by the grants of the Thematic Research Program from Academia Sinica, Taiwan, Republic of China (AS 103-TP-B11). The confocal microscopy was performed at the Advanced Optical Microscope Core Facility funded by the Scientific Instrument Center of Academia Sinica (AS-CFII-108-116).

## Conflict of Interest Statement

The authors declare that the research was conducted in the absence of any commercial or financial relationships that could be construed as a potential conflict of interest.

## Abbreviations

*At*PHT1;1, *Arabidopsis thaliana* phosphate transporter 1;1; TMs, transmembrane helices; MFS, major facilitator superfamily.
