# Supplementary material for: Structure–Function Analysis Reveals Amino Acid Residues of Arabidopsis Phosphate Transporter AtPHT1;1 Crucial for Its Activity
Source: Front Plant Sci. 2019 Sep 19;10:1158. doi: 10.3389/fpls.2019.01158 (PMC6761603; doi:10.3389/fpls.2019.01158)
Supplement: Supplementary file 2 [file Presentation_1.pdf]

Supplementary material

Structure-Function Analysis Reveals Amino Acid Residues of *Arabidopsis* Phosphate Transporter *At*PHT1;1 Crucial for Its Activity

Ya-Yun Liao[1], Jia-Ling Li[2], Rong-Long Pan[1,3] and Tzyy-Jen Chiou[2,3*]

[1] Department of Life Science, Institute of Bioinformatics and Structural Biology, College of Life Science, National Tsing Hua University, Hsin Chu 30013, Taiwan, Republic of China

[2] Agricultural Biotechnology Research Center, Academia Sinica, Taipei 115, Taiwan, Republic of China

[3] Co-corresponding authors.

***Correspondence:**

Dr. Tzyy-Jen Chiou

E-mail address: tjchiou@gate.sinica.edu.tw

Dr. Rong-Long Pan

E-mail address: rlpan@life.nthu.edu.tw

Keywords: Phosphate transporter, *At*PHT1;1, *Arabidopsis thaliana*, *Saccharomyces cerevisiae*, Topology, Site-directed mutagenesis, Major facilitator superfamily

Table S1. List of primer sequences.

Table S2. The corresponding amino acid residues in *At*PHT1;1, *Sc*PHO84, and *Pi*PT.

Table S3. Homologous residues of A-motif in YajR, XylE, *Pi*PT, and *At*PHT1;1.

Figure S1. Sequence alignment of the *At*PHT1;1 homologues.

Figure S2. Putative hydrogen bonds in the inward facing occluded state of *At*PHT1;1.

Figure S3. Prediction of intrinsically disordered regions in *At*PHT1;1.

Figure S4. Complementation analysis of *pht1;1* mutants.

Figure S5. Immunoblot analysis of *At*PHT1;1 variants expressed in yeast *pam2*.

Figure S6. Immunoblot analysis of *At*PHT1;1 variants expressed in *Arabidopsis pht1;1* mutants.

Figure S7. Plasma membrane localization of YFP-tagged *At*PHT1;1 variants expressed in tobacco leaves.

Data S1. Original and relative Pi contents of individual T2 lines analyzed in Figure 4B, 5B and 6B. (separate file)

Table S1. List of primer sequences.

The underlines show nucleotides encoding the amino acids were replaced to Alanine.

Name	Sequence (5’ to 3’)
*At*PHT1;1	F- CCCAAGCTTATGGCCGAACAACAACTAGG
*At*PHT1;1	R-AGGTTAGCCATGACGAGAAAGGTACCCATCATCATCATCAT CATTAATCTAGAGC
K16A	CTCGATGTTGCGGCCACGCAACTTTATC
D35A	GGTTTCTTTACCGCCGCCTACGATC
D38A	CCGATGCCTACGCCCTTTTTTGCGTG
D93A	GTTGGCTCGGTGCCAAACTCGGACG
K99A	CTCGGACGGAAAGCCGTGTACGGTCTC
E122A	CTTTTGGCCACGCCGCCAAGGGTGTC
K124A	GCCACGAAGCCGCCGGTGTCATGAC
R134A	CTTTGCTTCTTCGCCTTTTGGTTGGG
D144A	GTATTGGAGGTGCCTACCCACTTTC
Y145A	GGTATTGGAGGTGACGCCCCACTTTCTGCC
F169A	CGCAGCTGTCGCCGCCATGCAAG
Q172A	GTCTTCGCCATGGCCGGTGTCGGTATC
D191A	CTTCTATATTCGCCAAAAAGTTCCCAG
D212A	CTCCTCAAGTTGCCTACATTTGGCG
R216A	GACTACATTTGGGCCATCATCGTCATG
R233A	CTTACTACTGGGCCATGAAGATGCCTG
K235A	CTGGCGTATGGCCATGCCTGAAACTG
W304A	GAACTACCTCCACAGCCTTTTTGCTTGACATTGC
D308A	GGTTTTTGCTTGCCATTGCCTTCTAC
Y312A	CTTGACATTGCCTTCGCCAGCCAAAACTTGTTC
R345A	GTTTTCAGGATTGCTGCCGCTCAGACTCTTATC
D367A	GTTGCGTTTATTGCCACCATTGGAAG
K373A	CATTGGAAGGTTTGCCATCCAACTAAATGG
R402A	CAAACCAGAAAACGCCATCGGATTTGTGG
N421A	CGCCAATTTTGGTCCAGCCGCAACCACTTTTATTG
K449A	GCCGCGGCTGGTGCCGCTGGAGCCATTG
K482A	GGCATCGGAGTTGCCAACTCATTGATC
E504A	CTTCCTTGTCCCAGCCCCCAAAGGCAAG

Table S2. The corresponding amino acid residues in *At*PHT1;1, *Sc*PHO84, and *Pi*PT.

Proposed function in *Pi*PT	Transmembrane	*Pi*PT	*Sc*PHO84	*At*PHT1;1
Phosphate binding site	TM4	Y150	Y179	Y145
Phosphate binding site	TM5	F174	F203	F169
Phosphate binding site	TM5	Q177	Q206	Q172
Phosphate binding site	TM7	W320	W354	W304
Phosphate binding site	TM7	D324	D358	D308
Phosphate binding site	TM7	Y328	Y362	Y312
Phosphate binding site	TM10	N431	N464	N421
Phosphate binding site (?)	TM11	K459	K492	K449
Proton transfer	TM1	D45	D76	D35
Proton transfer	TM1	D48	D79	D38
Proton transfer	TM3	E108	E138	-
Proton transfer	TM4	R139	R168	R134
Proton transfer	TM4	D149	D178	D144

? Indicates K459 has no directly interaction with Pi in the inward occluded state of *Pi*PT (Pedersen et al., 2013)

- No conserved residue in *At*PHT1;1

Table S3. Homologous residues of A-motif in YajR, XylE, *Pi*PT, and *At*PHT1;1.

	YajR	XylE	*Pi*PT	*At*PHT1;1
Gating homologous residues				
**Outward open state**				
● TM2-TM3 Loop	*D73-R77		D97-R101	D93-R97
**Outward-facing occluded state**				
● TM2-TM3 Loop/TM4/TM11		*R84-E153-R404	R101-D159-R447	R97-E154-R437
● TM5/TM8-TM9 Loop/ TM10-TM11 Loop		*R160-D337-R341-E397	R166-D381-R385-E440	R161-D367-R371-E430
**Inward-facing occluded state**				
● TM4-TM5 Loop/TM8			*R165-D381	R161-D367
● TM4-TM5 Loop/TM11		E153-R404	*D159-R447	E154-R437
● TM4-TM5 Loop/ TM10-TM11 Loop		R160-E397	*R166-E440	R161-E430
**Inward open state**				
● TM2-TM3 Loop/TM4		*R84-E153	R101-D159	R97-E154
● TM8-TM9 Loop/ TM10-TM11 Loop		*R341-E397	R385-E440	R371-E430

*indicates the formation of salt bridges among residues found in YajR, XylE, *Pi*PT, respectively (Sun et al., 2012; Jiang et al., 2013; Pedersen et al., 2013; Wisedchaisri et al., 2014; Quistgaard et al., 2016).

Underline shows A-Motif residues (TM2-TM3/TM8-TM9; GX$_3$DRXXRR sequence)

- indicates the electrostatic interactions among residues

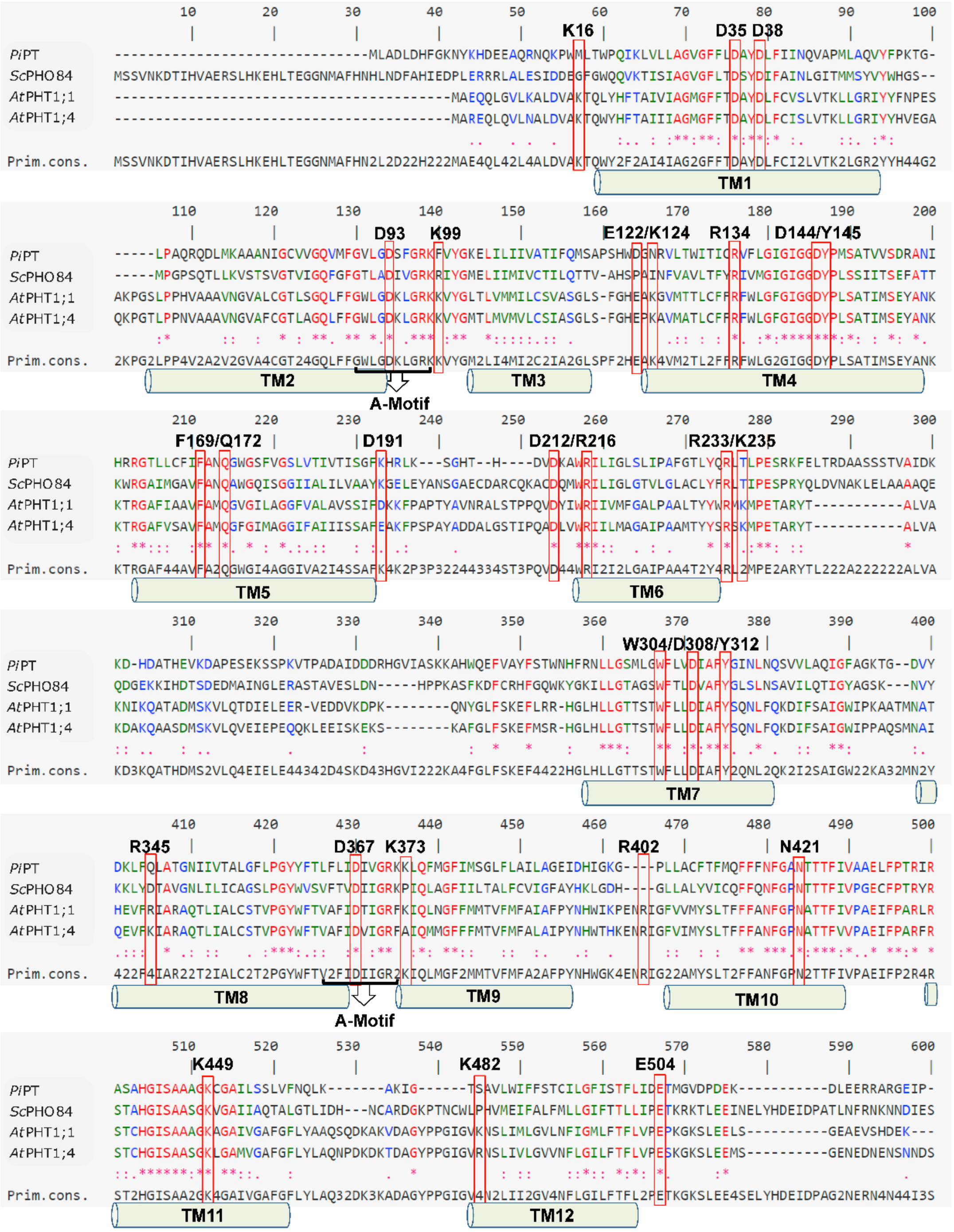

Figure S1. Sequence alignment of the *At*PHT1;1 homologues.

Accession number: *At*PHT1;1 (Q8VYM2) and *At*PHT1;4 (Q96303) from *A. thaliana*, *Sc*PHO84 (P25297) from *S. cerevisiae*, and *Pi*PT (A8N031) from *P. indica*. Protein sequences were aligned using ClustalW (PBIL)

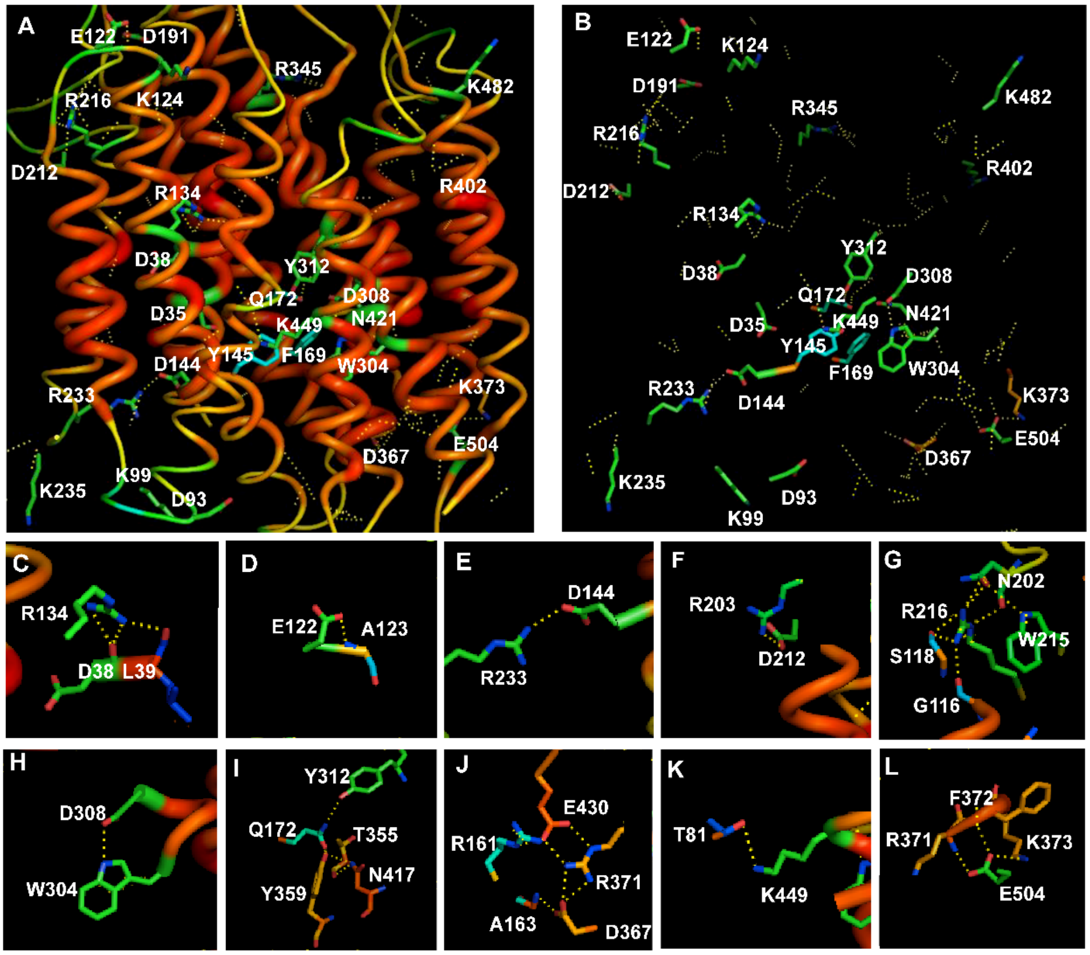

Figure S2. Putative hydrogen bonds in the inward facing occluded state of *At*PHT1;1. Putative hydrogen bonds within the side chains of 27 amino acid residues were examined (without K16), as illustrated together with (A) or without (B) TMs and loops. The putative hydrogen bonds within the main chains (MC) or side chains of several residues are indicated: (C) R134-D38(MC)-L39(MC); (D) E122-A123(MC); (E) D144-R233; (F) D212-R203; (G) R216-N202-W215-S118(MC)-G116(MC); (H) W304-D308; (I) Q172-Y312-Y359-N417-T355(MC); (J) D367-R371-E430-R161-A163(MC); (K) K449-T81; (L) K373-E504-F372(MC)-R371(MC). Yellow dots represent hydrogen bonds. These structure figures were prepared by PyMol.

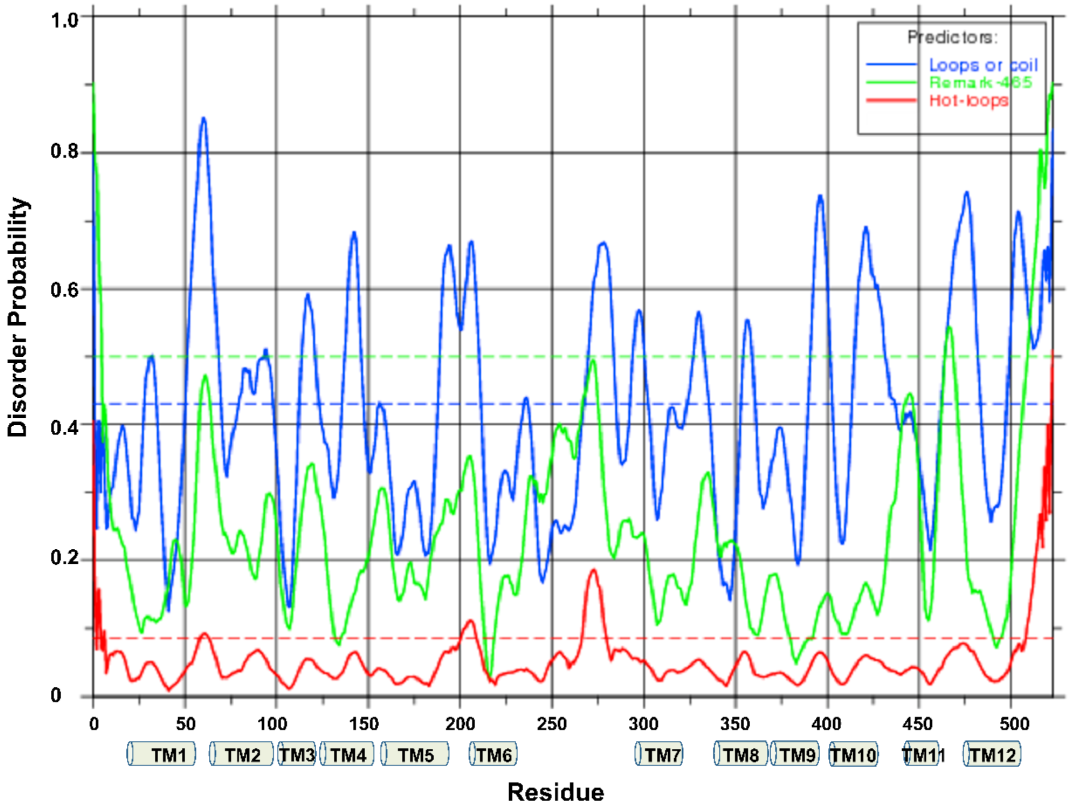

Figure S3. Prediction of intrinsically disordered regions in *At*PHT1;1.

Amino acid residues predicted to be in the disordered loop/coils regions are: I52-A70, A114-A123, L137-S148, I189-D212, E269-F285, G294-T303, I325-A336, S354-F361, F391-I403, F415-R437, A463-S484, and T499-K524. Blue line, loop/coils represent regular secondary structure and the threshold values of loop/coils over 0.43 suggest the disordered residues. Amino acid residues predicted to be in the disordered hot-loops region are: E268-Q280 and L510-K524. Red line, hot loops indicate highly mobile loops and the threshold values of hot-loops over 0.09 suggest the disordered residues. Disordered amino acid residues accepted by REMARK-465 are L510-K524. Green line represents the region lacking electron density in crystal structure and the threshold values of loop/coils over 0.50 suggest the disordered residues.

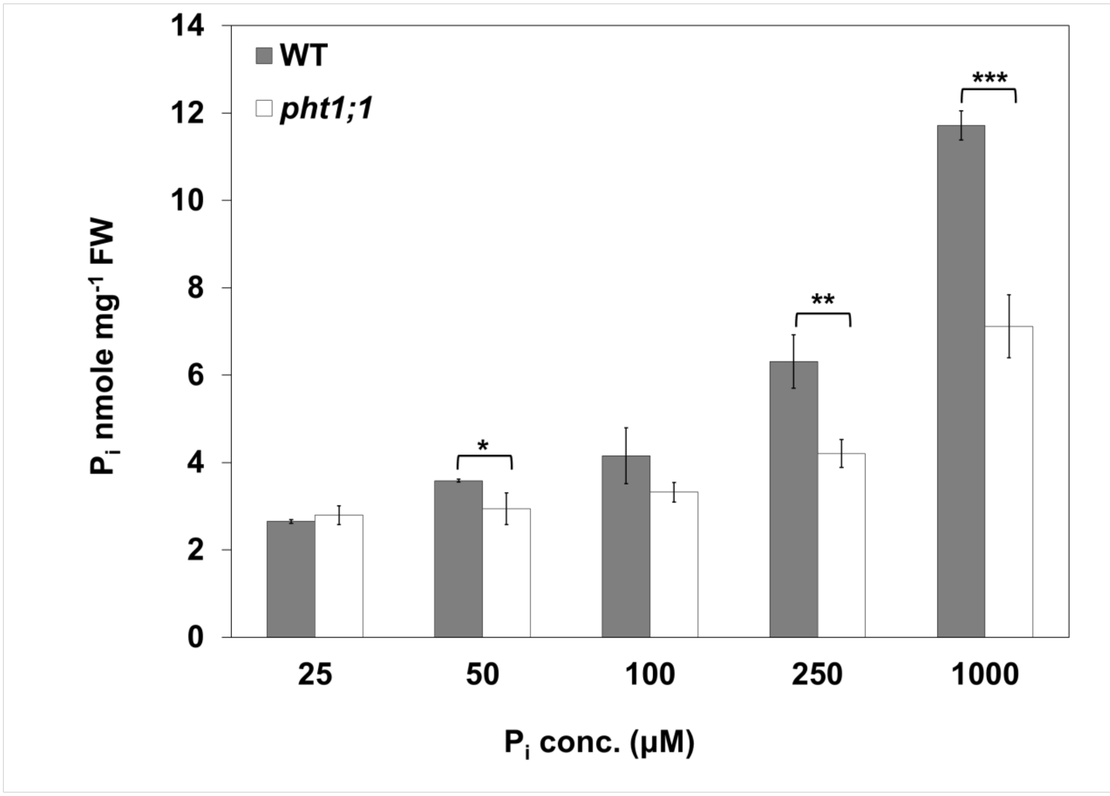

Figure S4. Complementation analysis of *pht1;1* mutants.

Five-day-old *Arabidopsis* seedlings of WT and *pht1;1* grown in 250 µM KH_2PO_4 media were transferred to media supplemented with different concentrations of KH_2PO_4 for another 7 days. Seedlings were collected for the measurement of Pi content. Error bars represent SD (n = 3). Asterisks indicate a significant difference compared with WT. *$P < 0.05$, **$P < 0.01$, ***$P < 0.001$, student's t-test.

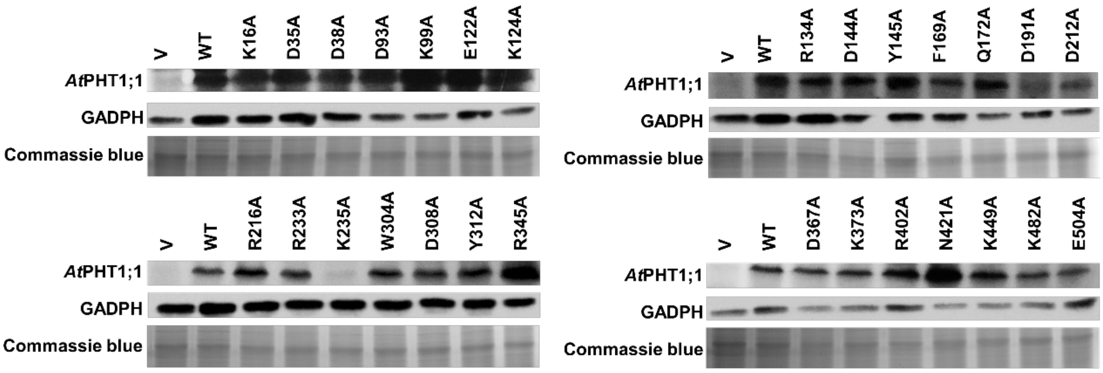

Figure S5. Immunoblot analysis of *At*PHT1;1 variants expressed in yeast *pam2*.

Total proteins stained by Coomassie Blue and GAPDH detected by the anti-GAPDH antibody served as loading controls.

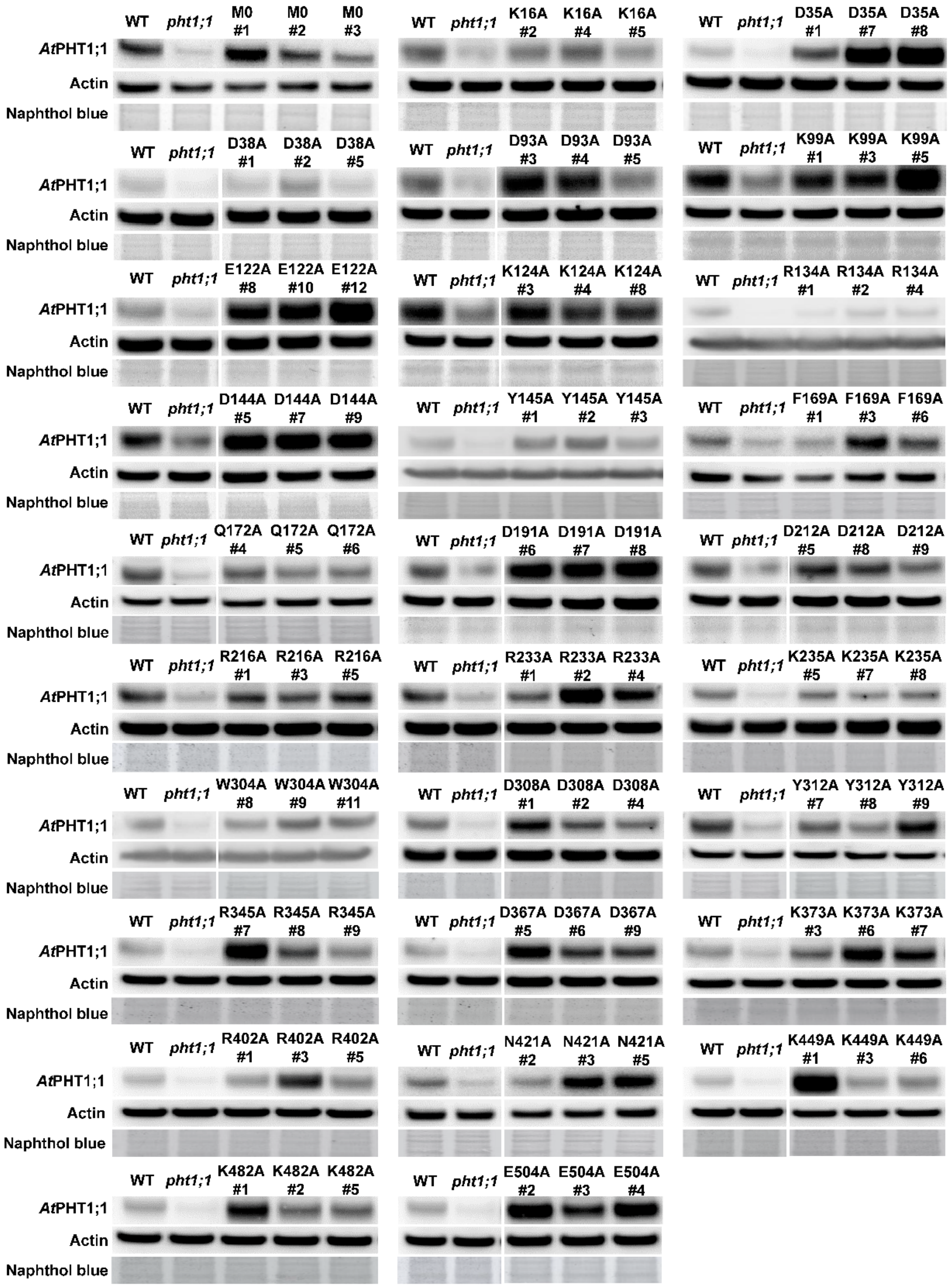

Figure S6. Immunoblot analysis of *At*PHT1;1 variants expressed in *Arabidopsis pht1;1* mutants.

For each variant, 3 independent transgenic lines are analyzed. Actin detected by the anti-Actin antibody served as a loading control. The following pairs of variants were run on the same blot for analysis and thus shared the same controls of WT and *pht1;1*: K16A and D93A, D35A and D38A, K99A and K124A, D191A and D212A, R216A and R233A, K235A and D308A, R345A and D367A, K373A and E122A, R402A and K449A, and K482A and E504A.

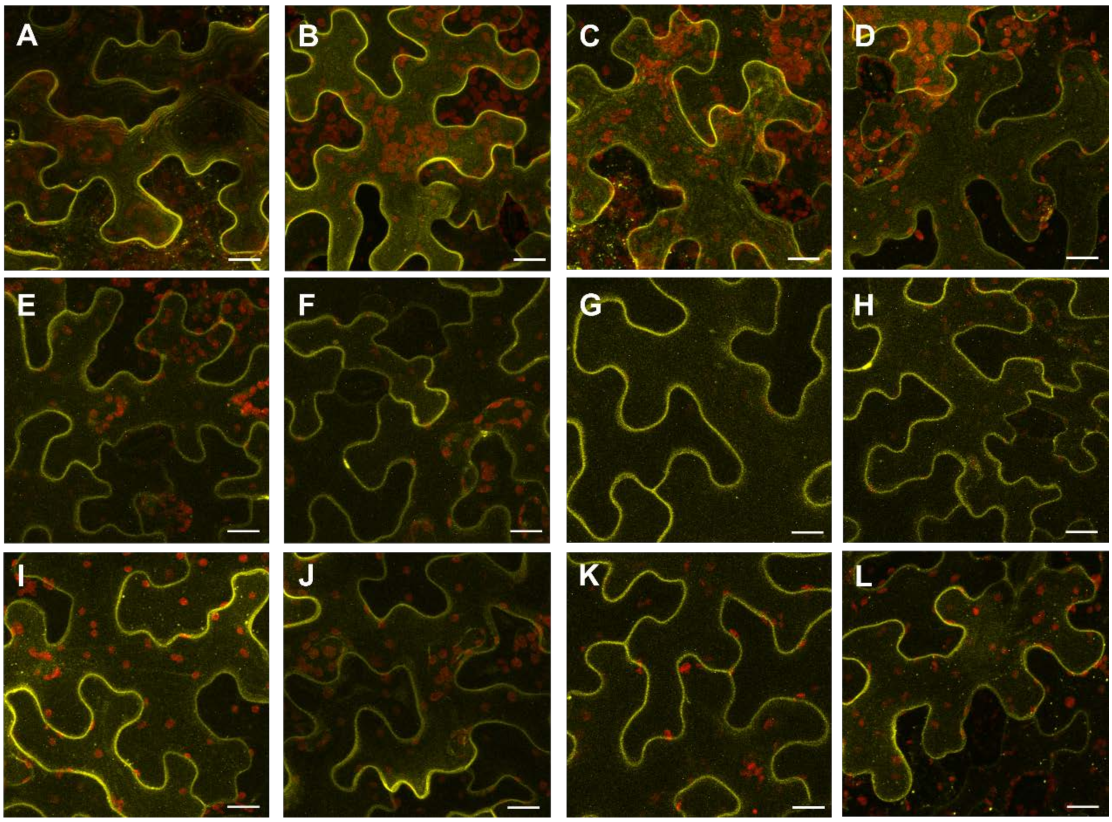

Figure S7. Plasma membrane localization of YFP-tagged *At*PHT1;1 variants expressed in tobacco leaves.

(A) M0, (B) D35A, (C) D93A, (D) D144A, (E) D212A, (F) R216A, (G) R233A, (H) R345A, (I) D367A, (J) K373A, (K) R402A, and (L) K449A, Bars = 20 μm.
